# AL Amyloidosis: The Effect of Maintenance Therapy on Autologous Stem Cell Transplantation Outcomes

**DOI:** 10.3390/jcm9113778

**Published:** 2020-11-23

**Authors:** Michael Ozga, Qiuhong Zhao, Don Benson, Patrick Elder, Nita Williams, Naresh Bumma, Ashley Rosko, Maria Chaudhry, Abdullah Khan, Srinivas Devarakonda, Rami Kahwash, Ajay Vallakati, Courtney Campbell, Samir V. Parikh, Salem Almaani, Jason Prosek, Jordan Bittengle, Katherine Pfund, Samantha LoRusso, Miriam Freimer, Elyse Redder, Yvonne Efebera, Nidhi Sharma

**Affiliations:** 1 Division of Hematology, Department of Internal Medicine, The Ohio State University Comprehensive Cancer Center, Columbus, OH 43210, USA; michael.ozga@osumc.edu (M.O.); qiuhong.zhao@osumc.edu (Q.Z.); Don.benson@osumc.edu (D.B.); patrick.elder@osumc.edu (P.E.); nita.williams@osumc.edu (N.W.); naresh.bumma@osumc.edu (N.B.); Ashley.Rosko@osumc.edu (A.R.); maria.chaudhry@osumc.edu (M.C.); Abdullah.Khan@osumc.edu (A.K.); srinivas.devarakonda@osumc.edu (S.D.); Jordan.Bittengle@osumc.edu (J.B.); Katherine.Pfund@osumc.edu (K.P.); yvonne.efebera@osumc.edu (Y.E.); 2 Division of Cardiology, Department of Internal Medicine, The Ohio State University, Columbus, OH 43210, USA; rami.kahwash@osumc.edu (R.K.); ajay.vallakati@osumc.edu (A.V.); courtney.campbell@osumc.edu (C.C.); 3 Division of Nephrology, Department of Internal Medicine, The Ohio State University, Columbus, OH 43210, USA; Samir.Parikh@osumc.edu (S.V.P.); salem.almaani@osumc.edu (S.A.); Jason.Prosek@osumc.edu (J.P.); 4 Department of Neurology, The Ohio State University, Columbus, OH 43210, USA; Samantha.LoRusso@osumc.edu (S.L.); Miriam.Freimer@osumc.edu (M.F.); 5 Department of Oncology Rehabilitation, The Ohio State University, Columbus, OH 43210, USA; Elyse.Redder@osumc.edu

**Keywords:** AL amyloidosis, transplantation, maintenance therapy, fluorescent in situ hybridization, plasma cell burden

## Abstract

Background: Autologous stem cell transplantation (ASCT) remains an effective treatment option for many patients with systemic light chain (AL) amyloidosis. While maintenance post ASCT in multiple myeloma is now standard, the decision to utilize maintenance in AL amyloidosis remains largely unexplored. The present study aims to determine the prognostic significance of utilizing maintenance therapy following ASCT and assess the impact of fluorescent in situ hybridization (FISH) abnormalities, bone marrow plasma cell burden (BMPC), and degree of organ involvement on this decision. Methods and results: This is a retrospective analysis of fifty AL amyloidosis patients who underwent ASCT at The Ohio State University. Twenty-eight patients received maintenance and twenty-two did not. Kaplan–Meier survival analysis was used to compare the effect of maintenance therapy with no significant difference in PFS (*p* = 0.66) and OS (*p* = 0.32) between the two groups. There was no difference in survival based on maintenance when further categorized by FISH, PFS (*p* = 0.15), and OS (*p* = 0.65); BMPC ≥ 10%, PFS (*p* = 0.49), and OS (*p* = 0.32); or with 2 or more organs involved, PFS (*p* = 0.34) and OS (*p* = 0.80). Conclusion: Maintenance therapy post ASCT did not impact PFS or OS when categorized by FISH abnormalities, increasing BMPC, or ≥2 organs involved in AL amyloidosis patients.

## 1. Introduction

Systemic immunoglobulin light chain (AL) amyloidosis is a rare clonal plasma cell neoplasm characterized by deposition of fragments of light chain in tissues [1,2,3]. AL amyloidosis has a reported incidence of 6–9 cases per million person-years in the United States [4,5], approximately one-tenth that of multiple myeloma, a related plasma cell disorder. Despite the paucity of cases seen in the United States, the past two decades have seen an emergence in treatment options for these often-complex patients, including chemotherapy regimens with immunomodulatory agents and proteasome inhibitors that work to eliminate amyloidogenic proteins, prevent fibril formation and deposition, and ameliorate markers of disease. To this end, the utility of autologous stem cell transplantation (ASCT) in this arena has also remained an effective therapy regimen for many patients with AL amyloidosis. This dates back to an instrumental study done in 1998 [6] that enrolled 25 patients with AL amyloidosis to receive melphalan 200 mg/m^2^ followed by ASCT. ASCT was also most recently discussed by our group [7], who summarized the use of dose-adjusted melphalan and ASCT as a safe and reasonable option in this setting. However, the decision to utilize maintenance therapy following transplant remains controversial and largely unexplored. Studies have shown the survival benefit of post-ASCT maintenance therapy with lenalidomide in multiple myeloma (MM) [8], but to date, we know of no study that has evaluated maintenance therapy in AL amyloidosis patients following transplant. Moreover, our group and others have previously reported on the growing relevance of fluorescent in situ hybridization (FISH) abnormalities, bone marrow plasmacytosis, and degree of organ involvement in AL amyloidosis [9,10], which may also help dictate the incorporation of maintenance therapy. The present study thus aims to determine the prognostic significance of utilizing maintenance therapy following ASCT and assess the potential impact of FISH markers, bone marrow plasma cell burden (BMPC), and degree of organ involvement on the efficacy of maintenance.

## 2. Patients and Methods

### 2.1. Patient Population

This is a retrospective study analyzing 50 consecutive newly diagnosed AL amyloidosis patients who underwent ASCT at the Ohio State University (OSU) between 2001 and 2019. The study was approved by the institutional review board at OSU and follows the principles of the Declaration of Helsinki. Patients were divided according to whether or not they received maintenance therapy following ASCT, defined as therapy longer than six months following ASCT. The decision to utilize maintenance therapy was largely determined by physician discretion at our center.

### 2.2. FISH Abnormalities

CD138-enriched chromosome-specific FISH panels for MM obtained from bone marrow aspirate samples were studied. FISH enumeration strategies were utilized to detect monosomies (deletions) or gains (trisomies/tetrasomies) of the following chromosomes with respective probe sets: 17 (17p13.1 and 17q21), 13 (13q14 RB1 and 13q34 LAMP1), 3 (D3Z1), 6 (6q21), 5 (5q33-34,5p152 (CSF1R-D5S23:D5S721)), 19 (19p13 TCF), 12 (12p13 ETV6 and 12cen), 1 (both 1q21 CKS1B/1q23 PBX1 and 1p CHD5), 11 (both 11q13 CCND1 and 11q23 ATM), 9 (D9Z1), and 15 (D15Z4). We also analyzed translocations involving the immunoglobulin heavy chain (IgH) and several partners, most notably 11q13 (CCND1), followed by 4p16.3 (FGFR3), 16q23 (MAF), 20q12 (MAFB), and 6p21 (CCND3). Patients were divided into subgroups based on the above FISH data, including a binary assessment of abnormal (presence of any abnormality) versus normal FISH samples.

### 2.3. Data Collection

All laboratory values were collected at the time of diagnosis and recorded in the electronic medical record. Patient variables assessed included dose of melphalan conditioning received prior to ASCT (140 or 200 mg/m^2^, dose adjusted secondary to age and renal function if required), presence of FISH abnormalities obtained within 90 days of diagnosis, and degree of BMPC, with ≥10% defined as the cutoff for higher plasmacytosis and indicating either coexistent smoldering multiple myeloma (SMM) or MM based on International Myeloma Working Group consensus criteria [11]. Kappa (κ) and lambda (λ) light chain restriction was recorded as well as the resultant κ/λ ratio and difference between involved and uninvolved light chain (dFLC). Organ involvement was delineated into “cardiac”, “kidney”, “hepatic”, “gastrointestinal”, “peripheral neuropathy”, or “other” (i.e., soft tissue) categories, based on tissue biopsies demonstrating apple green birefringence under polarized light, in conjunction with related serum biomarkers and imaging studies as previously established via consensus from the International Society of Amyloidosis [1,12]. Higher degree of AL amyloidosis organ involvement was defined as ≥2 organs with amyloid deposition at diagnosis. Patients were staged primarily based on guidelines presented by the Mayo Clinic group, both the 2004 and 2012 serum biomarker systems [13,14].

### 2.4. Statistical Analysis

Patient characteristics were summarized using median and range for continuous variables, and frequency and percentage for categorical variables. The comparison of patient characteristics between groups were conducted using Wilcoxon rank-sum (Mann–Whitney) test for continuous variables and Fisher’s exact or chi-square test for categorical variables, whichever was appropriate. Primary endpoints were progression-free survival (PFS) and overall survival (OS), per updated NCCN guidelines [3] including both organ and hematologic criteria. PFS was calculated from the date of transplant to the date of disease progression or death from any cause, censoring those who did not progress at the last clinical assessment date. OS was defined as the time from date of transplant to death from any cause, censoring those who were still alive at the date of last follow up. Kaplan–Meier survival function was used to estimate the PFS and OS with log-rank analysis used to test the equality of survivor functions between different groups of patients. Cox proportional hazard model was used to estimate the risk of relapse/death. The significance level was set at α = 0.05 and all *p*-values presented are from two-sided tests. The statistical analysis was performed using Stata 14.

## 3. Results

### 3.1. Patient Characteristics

Baseline demographics and clinical characteristics of the fifty AL patients who underwent ASCT are in Table 1. The median age at diagnosis was 58 years (range: 33–71) and 66% were male. The majority of patients (50%) possessed primary systemic light-chain amyloidosis, with 14 patients (28%) having concomitant SMM and 11 (22%) with concomitant MM. Of the entire cohort, seventy percent of patients had λ light chain (LLC) clonal disease. The median time from diagnosis to ASCT was 184 days. Regarding induction regimens received, the majority of patients received bortezomib (velcade)-based regimens, with 34% (17/50) receiving either velcade–dexamethasone (Vd) or velcade–lenalidomide–dexamethasone (VRD). Ten patients (20%) did not receive induction therapy and proceeded directly to ASCT. The majority of patients (54%) received high-dose melphalan, 200 mg/m^2^, as part of their conditioning regimen prior to ASCT. The majority of patients (47/50) had FISH cytogenetics completed at diagnosis, with thirty patients (60%) possessing at least one abnormal FISH result. Eighteen patients (38%) were found to have a *t*(11;14) mutation. Twenty-eight patients received maintenance and twenty-two did not, with the median duration of maintenance therapy of 2.27 years. The majority of patients (86%) received immunomodulatory (IMiD)-based maintenance therapy, primarily with lenalidomide, with the remainder receiving bortezomib-based regimens. Twenty-six patients (52%) had BMPC ≥10%, in which 77% (20/26) received maintenance therapy, which is significantly higher compared to 33% (8/24) of those patients with ≤10% BMPCs (*p* = 0.004). The median number of organs involved was two, with 54% of patients having ≥2 organs involved at diagnosis, and 36% and 80% having cardiac and kidney involvement, respectively. Thirteen patients also possessed nerve involvement (26%), followed by gastrointestinal involvement (24%), and hepatic involvement (14%). There was no difference in age (*p* = 0.99), dose of melphalan used (*p* = 0.53), disease staging (based on Mayo 2012 staging systems) (*p* = 0.460), and number of organs involved between the two groups (*p* = 0.91) (Table 1). 

### 3.2. Hematological/Organ Response and Survival/Prognosis Outcomes

**Figure 1:**
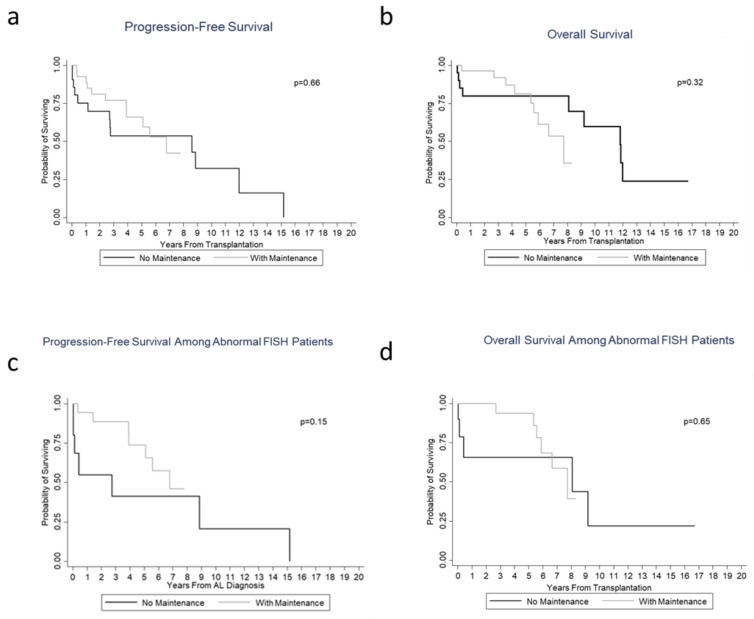
Kaplan–Meier curves demonstrating survival measured from maintenance (**a**) Progression-free survival of patients with or without maintenance. (**b**) Overall survival of patients with or without maintenance. (**c**) Progression-free survival among patients with abnormal FISH with or without maintenance. (**d**) Overall survival among patients with abnormal FISH with or without maintenance.

**Figure 2:**
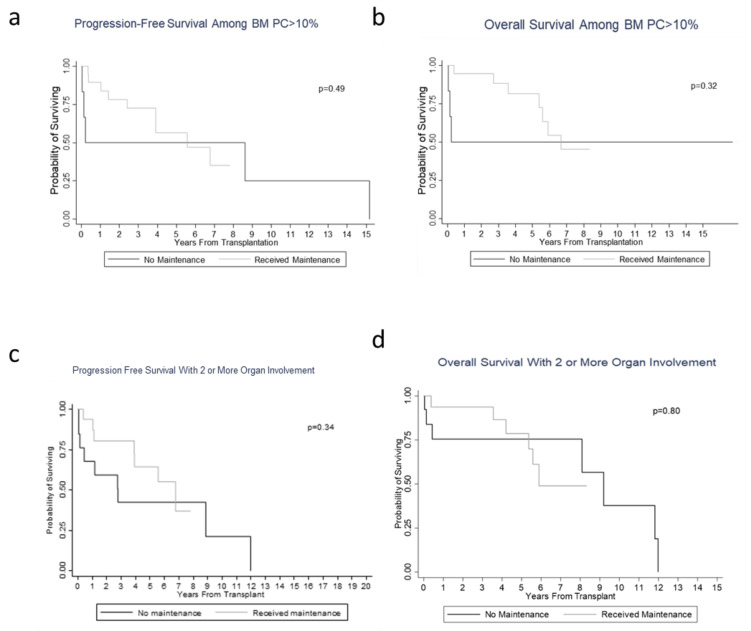
Kaplan–Meier curve demonstrating survival measured from maintenance. (**a**) Progression-free survival among patients with ≥10% plasma cell burden with or without maintenance. (**b**) Overall survival among patients with ≥10% plasma cell burden with or without maintenance. (**c**) Progression-free survival among patients with ≥2 organs with or without maintenance. (**d**) Overall survival among patients with ≥ 2 organs with or without maintenance.

Twenty-two patients (44%) experienced a hematological complete response (hCR) following ASCT at first follow up within three months, followed by 19 (38%) with very good partial response (hVGPR), 5 (10%) with partial response (hPR), and 4 (8%) with stable disease (hSD). Specifically looking at organ involvement with heart and renal amyloid disease, the majority of cardiac AL patients (66%) experienced a partial response with only 1 patient with cardiac CR, and renal patients trended toward SD responses (66%), with the highest level of response noted as a PR. Patients that received maintenance therapy following ASCT did not experience further deepened levels of hematological or organ responses, with 7 patients (7/28, 25%) having progression of disease following initiation of maintenance therapy. Despite the relatively high percentage of patients achieving hCR following ASCT, when further analyzing survival outcomes, there was no statistical difference in PFS and OS between the maintenance and non-maintenance groups. The median follow-up time was 4.53 (0.27–8.33) years in the maintenance group and 5.71 (0.03–16.73) years in the no maintenance group. The median PFS for patients with maintenance was 6.8 years (95% CI: 3.9–not reached (NR)) compared to 2.8 years (95% CI: 0.2–12.0 years) for patients without maintenance (*p* = 0.66), and the median OS was 7.7 years (95% CI: 5.6–NR) versus 11.8 years (95% CI: 0.4–12.0 years) (*p* = 0.32), respectively (Figure 1a,b). Specifically, among patients with abnormal FISH, there was also no difference in survival between these two groups in PFS (*p* = 0.15) and OS (*p* = 0.65) (Figure 1c,d). Moreover, when analyzing patients with higher BMPC at diagnosis (*n* = 26 patients), the decision to utilize maintenance therapy versus no maintenance did not make a difference in terms of PFS and OS (Figure 2a,b). Thus, even though there was a difference in maintenance therapy administration between those patients with higher BMPCs compared to their lower BMPCs counterparts, there was no impact on survival. Upon further stratification by degree of organ involvement, i.e., patients with 2 or more organs involved, the decision to utilize maintenance vs. no maintenance showed no difference in PFS (*p* = 0.34) and OS (*p* = 0.80) (Figure 2c,d).

### 3.3. Toxicity Profiles in the Maintenance Group

Of the 28 patients exposed to maintenance therapy, seven patients (7/28, 25%) experienced progression of disease, necessitating cessation of maintenance therapy and subsequent resumption of treatment-based therapy. Maintenance therapy was also discontinued in four patients (4/28, 14%) due to myelosuppression toxicities, including profound thrombocytopenia and infection-related adverse events. Moreover, of our patient cohort that received maintenance lenalidomide, four of these patients (4/28, 14%) suffered cardiac-related causes of death, including pulseless electrical activity (PEA) arrest and non-ST elevated myocardial infarction (NSTEMI). These patients did have underlying pre-existing cardiac amyloid disease, but we did not observe a subsequent increase in NT-proBNP in these specific cases to suggest overt cardiac progression at that time. There were also no secondary maintenance-therapy-related malignancies to report. The following were the causes of death in both maintenance and non-maintenance therapy groups: therapy-related infections (2/28 in maintenance vs. 4/22 in non-maintenance), cardiac-related causes of death as above (4/28 vs. 2/22), disease progression (1/28 vs. 2/22), and overall failure to thrive/hospice-related outcomes (4/28 vs. 3/22). Regarding financial toxicities, the average wholesale price per lenalidomide capsule is $916; however, the financial burden incurred by patients on maintenance therapy varied widely due to insurance coverages and grant charitable donations. The socioeconomic responsibility in working with insurance companies and affording co-pays remains, however, with each monthly installment of maintenance therapy.

## 4. Discussion

In our cohort of 50 newly diagnosed AL patients who underwent ASCT, over half of our patients (56%) received maintenance therapy following transplant. However, despite the survival benefit seen in multiple myeloma with lenalidomide, we did not observe a difference in PFS or OS in AL amyloidosis patients exposed to maintenance therapy following transplant, despite a high percentage of hCR. This is potentially secondary to the oft-delayed organ responses experienced by these patients and as evidenced in our patient cohort. Moreover, upon stratification by FISH abnormalities, plasma cell burden, and degree of organ deposition, there was also no survival benefit obtained with the addition of maintenance therapy. It is interesting to note, however, that the median PFS was four years longer in the maintenance subgroup, while median OS was four years longer in the non-maintenance subgroup. The reason for this is not explicitly clear, but may be related to our limited sample size and dataset, or potentially suggests that patients may derive minor immediate delay in progression of disease upfront with these maintenance therapies, but may ultimately incur cumulative side effects and toxicities related to their long-term use (i.e., cardiotoxicity from lenalidomide, long-term myelosuppression).

This study has limitations, largely owing to the retrospective and single-center nature of its analysis and the rare nature of this disease. We attempted to mitigate any selection bias by including all sequential patients treated at our center. Moreover, our numbers are limited by patients that actually received ASCT, with most providers at our center, excluding AL amyloidosis patients, staged III and IV according to the Mayo 2012 staging system. Importantly, our low sample size may not have had sufficient power to detect a difference between maintenance therapy and lack of maintenance therapy in those with higher bone marrow plasmacytosis. We showed a difference in maintenance therapy received between BMPC groups, but did not see a survival impact as those with higher BMPCs who received maintenance included 20 patients versus those without maintenance was only 6 patients. It remains to be seen with a larger sample size if there are a greater proportion of patients with higher bone marrow plasmacytosis that may benefit from maintenance therapy following ASCT in the future. We also were limited in our review of MRD status of patients who achieved a hCR given the time frame studied in our cohort as well as sample size. We do not have any quality of life assessments on these patients but certainly acknowledge the financial toxicities experienced by those on maintenance therapy as above.

Moving forward, recent studies published by Landau et al. [15,16] discussed the use of velcade–dexamethasone before and after ASCT as consolidative therapy that resulted in impressive overall hematological response rates and continued organ improvement. As such, this suggests a possibility of utilizing Vd as consolidation prior to maintenance therapy. This study showed that each treatment phase deepened the response, which is promising in its own right, but potentially suggests that maintenance following consolidative Vd would do the same or at least maintain remission. As we observed in our study, careful consideration of efficacy and toxicity of cumulative side effects would ultimately need to be taken into consideration.

In summary, we recommend a large prospective study is ultimately needed to assess the benefit of maintenance therapy post ASCT in AL amyloidosis patients. The decision to utilize maintenance therapy and its corresponding duration is largely driven by physician discretion at our center. In the interim, with the data presented above, it is clear that greater clinical discretion is required to assess the utility of maintenance therapy on a case-by-case basis. Regardless, it remains an exciting time in AL amyloidosis transplantation, with the addition of novel small-molecule agents, including daratumumab, which has shown promise in this clinical setting [17,18]. The use of daratumumab has recently been expanded into trial at our center (Andromeda trial) [19] and continues to grow in both upfront pre-ASCT induction and in relapsed/refractory settings following transplant. We have preliminary, unpublished results for five patients who received daratumumab prior to ASCT, four of which were in combination with cyclophosphamide–bortezomib–dexamethasone-based therapy. All five of these patients’ toxic free light chains were reduced, with all obtaining a hematologic VGPR prior to proceeding to transplant. This offers an exciting option in combination therapies with ASCT and further prospective data is warranted in the coming years.

## Figures and Tables

**Table 1:** Baseline and demographic data of patients at diagnosis.

Characteristics	All Patients (*n* = 50)	No Maintenance Therapy (*n* = 22)	Maintenance Therapy (*n* = 28)	*p*-Value
Median age at diagnosis (range), years	58 (33–71)	58 (33–71)	58 (39–70)	0.990
Gender, male, *n* (%)	33 (66.0)	15 (68.2)	18 (64.3)	0.773
Specific induction regimen received, *n* (%)	Lenalidomide–dexamethasone 5 (10%) Melphalan–prednisone 4 (8%) Bortezomib–dexamethasone 15 (30%) Bortezomib–lenalidomide–dexamethasone 2 (4%) Cyclophosphamide–bortezomib–dexamethasone 14 (28%) None 10 (20%)			
Specific maintenance therapy used, *n* (%) Immunomodulator (lenalidomide, thalidomide), bortezomib		0 (0) 0 (0)	24 (86) 4(14)	
Melphalan dose received, 140 mg/m^2^ 200 mg/m^2^	23 (46.0) 27 (54.0)	11 (50.0) 11 (50.0)	12 (42.9) 16 (57.1)	0.620
Light chain restriction (kappa)	14 (28)	4 (18.2)	10 (35.7)	0.215
Light chain restriction (lambda)	35 (70)	18 (81.8)	17 (60.7)	0.131
dFLC mg/dla median (range)	17.9 (1209–7001)	15.9 (300–607)	25 (1209–7001)	0.640
BMPCb <10% ≥10%	24 (48%) 26 (52%)	16 (72.7) 6 (27.3)	8 (28.6) 20 (71.4)	0.004
Urine total protein, mg/24 h, median (range)	6849 (0–81,921)	10,308 (0–81,921)	3758 (0–22,500)	0.06
No. of involved organs, median (range)	2 (0–5)	1 (0–4)	2 (1–5)	0.910
Cardiac involvement, *n* (%)	18 (36.0)	10 (45.5)	8 (28.6)	0.217
Renal involvement, *n* (%)	40 (80.0)	19 (86.4)	21 (75.0)	0.319
NT-proBNP ≥ 332 ng/L, *n* (%)	19 (70.4)	8 (72.7)	11 (68.8)	0.824
NT-proBNP ≥ 1800 ng/L, *n* (%)	11 (40.7)	6 (54.5)	5 (31.3)	0.226
Mayo stage (2012), *n* (%)				0.460
I	13 (50.0)	5 (50.0)	8 (50.0)	
II	7 (26.9)	4 (40.0)	3 (18.8)	
III	2 (7.7)	0 (0.0)	2 (12.5)	
IV	4 (15.4)	1 (10.0)	3 (18.8)	
Missing	24	12	12	

Abbreviations: ASCT, autologous stem cell transplant; dFLC, difference between involved and uninvolved free light chains; Kappa nl 3.3–19.4 mg/L, Lambda nl 5.71–26.3 mg/L; *p* < 0.05; Stage I: none the following are elevated: troponin T ≥ 0.025 ng/mL and NT-ProBNP ≥ 1800 pg/mL and serum immunoglobulin free light chain difference ≥ 18 mg/dL; if any one parameter is high, then Stage II; if two parameters are high then Stage III; and if all three are elevated, then Stage IV.

## References

[1] Kyle R.A., Linos A., Beard C.M., Linke R.P., Gertz M.A., O’Fallon W.M., Kurland L.T. (1992). Incidence and natural history of primary systemic amyloidosis in Olmsted County, Minnesota, 1950 through 1989. Blood.

[2] Gertz M.A. (2014). Immunoglobulin light chain amyloidosis: 2014 update on diagnosis, prognosis, and treatment. Am. J. Hematol..

[3] National Comprehensive Cancer Network (2019). Systemic Light Chain Amyloidosis. Version 1.

[4] Merlini G., Seldin D.C., Gertz M.A. (2011). Amyloidosis: Pathogenesis and New Therapeutic Options. J. Clin. Oncol..

[5] Desport E., Bridoux F., Sirac C., Delbes S., Bender S., Fernandez B., Quellard N., Lacombe C., Goujon J.-M., Lavergne D. (2012). AL Amyloidosis. Orphanet J. Rare Dis..

[6] Comenzo R.L., Vosburgh E., Falk R.H., Sanchorawala V., Reisinger J., Dubrey S., O’Hara C. (1998). Dose-intensive melphalan with blood stem-cell support for the treatment of AL (amyloid light-chain) amyloidosis: Survival and responses in 25 patients. Blood.

[7] Pandit A., Wei L., Bustamante L., Elder P., Falk W. (2019). Improved Treatment Related Mortality in Patients with Primary Systemic Amyloidosis (AL Amyloidosis) Undergoing Autologous Hematopoietic Stem Cell Transplant (aHSCT). Arch. Hematol. Blood Dis..

[8] McCarthy P.L., Holstein S.A., Petrucci M.T., Richardson P.G., Hulin C., Tosi P., Bringhen S., Musto P., Anderson K.C., Caillot D. (2017). Lenalidomide Maintenance After Autologous Stem-Cell Transplantation in Newly Diagnosed Multiple Myeloma: A Meta-Analysis. J. Clin. Oncol..

[9] Warsame R., Kumar S.K., Gertz M.A., Lacy M.Q., Buadi F.K., Hayman S.R., Leung N., Dingli D., Lust J.A., Ketterling R.P. (2015). Abnormal FISH in patients with immunoglobulin light chain amyloidosis is a risk factor for cardiac involvement and for death. Blood Cancer J..

[10] Varga C., Comenzo R.L. (2018). High-dose melphalan and stem cell transplantation in systemic AL amyloidosis in the era of novel anti-plasma cell therapy: A comprehensive review. Bone Marrow Transplant..

[11] Rajkumar S.V., Dimopoulos M.A., Palumbo A., Blade J., Merlini G., Mateos M.-V., Kumar S., Hillengass J., Kastritis E., Richardson P. (2014). International Myeloma Working Group updated criteria for the diagnosis of multiple myeloma. Lancet Oncol..

[12] Gertz M.A., Comenzo R., Falk R.H., Fermand J.P., Hazenberg B.P., Hawkins P.N., Merlini G., Moreau P., Ronco P., Sanchorawala V. (2005). Definition of organ involvement and treatment response in immunoglobulin light chain amyloidosis (AL): A consensus opinion from the 10th International Symposium on Amyloid and Amyloidosis, Tours, France, 18–22 April 2004. Am. J. Hematol..

[13] Dispenzieri A., Gertz M.A., Kyle R.A., Lacy M.Q., Burritt M.F., Therneau T.M., Greipp P.R., Witzig T.E., Lust J.A., Rajkumar S.V. (2004). Serum Cardiac Troponins and N-Terminal Pro-Brain Natriuretic Peptide: A Staging System for Primary Systemic Amyloidosis. J. Clin. Oncol..

[14] Kumar S.K., Dispenzieri A., Lacy M.Q., Hayman S.R., Buadi F.K., Colby C., Laumann K., Zeldenrust S.R., Leung N., Dingli D. (2012). Revised Prognostic Staging System for Light Chain Amyloidosis Incorporating Cardiac Biomarkers and Serum Free Light Chain Measurements. J. Clin. Oncol..

[15] Landau H.J., Hassoun H.T., Rosenzweig M.A., Maurer M., Liu J., Flombaum C., Di Bello C., Hoover E.L., Riedel E., Giralt S. (2013). Bortezomib and dexamethasone consolidation following risk-adapted melphalan and stem cell transplantation for patients with newly diagnosed light-chain amyloidosis. Leukemia.

[16] Landau H., Lahoud O., Devlin S., Lendvai N., Chung D.J., Dogan A., Landgren C.O., Giralt S., Hassoun H. (2020). Pilot Study of Bortezomib and Dexamethasone Pre- and Post-Risk-Adapted Autologous Stem Cell Transplantation in AL Amyloidosis. Biol. Blood Marrow Transplant..

[17] Kaufman G.P., Schrier S.L., Lafayette R.A., Arai S., Witteles R., Liedtke M. (2017). Daratumumab yields rapid and deep hematologic responses in patients with heavily pretreated AL amyloidosis. Blood.

[18] Milani P., Merlini G., Palladini G. (2018). Novel Therapies in Light Chain Amyloidosis. Kidney Int. Rep..

[19] Palladini G., Kastritis E., Maurer M.S., Zonder J.A., Minnema M.C., Wechalekar A.D., Jaccard A., Lee H.C., Bumma N., Kaufman J.L. (2020). Daratumumab plus CyBorD for patients with newly diagnosed AL amyloidosis: Safety run-in results of ANDROMEDA. Blood.

